# The comprehensive profile of fermentation products during in situ CO_2_ recycling by Rubisco-based engineered *Escherichia coli*

**DOI:** 10.1186/s12934-016-0530-7

**Published:** 2016-08-02

**Authors:** Cheng-Han Yang, En-Jung Liu, Yi-Ling Chen, Fan-Yu Ou-Yang, Si-Yu Li

**Affiliations:** Department of Chemical Engineering, National Chung Hsing University, Taichung, 402 Taiwan

**Keywords:** The Calvin–Benson–Bassham (CBB) cycle, Ribulose-1,5-bisphosphate carboxylase/oxygenase (Rubisco), Pyruvate, Mixotroph

## Abstract

**Background:**

In our previous study, the feasibility of Rubisco-based engineered *E. coli* (that contains heterologous phosphoribulokinase (PrkA) and Rubisco) for in situ CO_2_ recycling during the fermentation of pentoses or hexoses was demonstrated. Nevertheless, it is perplexing to see that only roughly 70 % of the carbon fed to the bacterial culture could be accounted for in the standard metabolic products. This low carbon recovery during fermentation occurred even though CO_2_ emission was effectively reduced by Rubisco-based engineered pathway.

**Results:**

In this study, the heterologous expression of form I Rubisco was found to enhance the accumulation of pyruvate in *Escherichia coli* MZLF [*E. coli* BL21(DE3) Δ*zwf*, Δ*ldh*, Δ*frd*]. This may be attributed to the enhanced glycolytic reaction supported by the increased biomass and the ethanol/acetate ratio. Besides, it was found that the transcription of *arcA* (encodes the redox-dependent transcriptional activators ArcA that positively regulates the transcription of pyruvate formate-lyase) was down-regulated in the presence of Rubisco. The enhanced accumulation of pyruvate also occurs when PrkA is co-expressed with Rubisco in *E. coli* MZLF. Furthermore, *E. coli* containing Rubisco-based engineered pathway has a distinct profile of the fermentation products, indicating CO_2_ was converted into fermentation products. By analyzing the ratio of total C-2 (2-carbon fermentation products) to total C-1 (1-carbon fermentation product) of MZLFB (MZLF containing Rubisco-based engineered pathway), it is estimated that 9 % of carbon is directed into Rubisco-based engineered pathway.

**Conclusions:**

Here, we report for the first time the complete profile of fermentation products using *E. coli* MZLF and its derived strains. It has been shown that the expression of Rubisco alone in MZLF enhances the accumulation of pyruvate. By including the contribution of pyruvate accumulation, the perplexing problem of low carbon recovery during fermentation by *E. coli* containing Rubisco-based engineered pathway has been solved. 9 % of glucose consumption is directed from glycolysis to Rubisco-based engineered pathway in MZLFB. The principle characteristics of mixotroph MZLFB are the high bacterial growth and the low CO_2_ emission.

**Electronic supplementary material:**

The online version of this article (doi:10.1186/s12934-016-0530-7) contains supplementary material, which is available to authorized users.

## Background

The Calvin–Benson–Bassham (CBB) cycle, also known as the reductive pentose phosphate cycle, is a metabolic pathway involving the conversion of three molecules of carbon dioxide into one molecule of glyceraldehyde-3-phosphate. The CBB cycle uses 11 enzymes to complete autotrophic CO_2_ fixation. Interestingly, most of enzymes are also involved in the central metabolism including glycolysis and the pentose phosphate pathway. Therefore, it is generally accepted that the functional CBB cycle that emerged billions of years ago did not evolved as a whole, but instead the individual enzymes have their own phylogenetic history that is independent of the biochemical distribution of the CBB cycle [1]. Consequently, one way to genetically assess whether a microorganism utilizes the CBB cycle is to characterize the manifestation of two key genes that encodes phosphoribulokinase (PRK) and Ribulose-1,5-bisphosphate carboxylase/oxygenase (Rubisco). Rubisco is the one enzyme that is specific to the CBB cycle. Rubisco catalyzes the carboxylation and the oxygenation of ribulose-1,5-bisphosphate with CO_2_ and O_2_, respectively. The products of carboxylation and oxygenation are 3-phosphoglycerate and 2-phosphoglycolate and the former will be sequentially converted into glyceraldehyde-3-phosphate by phosphoglycerate kinase and glyceraldehyde-3 phosphate dehydrogenase whereas the latter will enter photorespiration [2].

The CBB cycle has been used in the engineering perspective for converting CO_2_ into bio-based chemicals. For example, microalgae have been used to produce carbohydrates from CO_2_, where the carbohydrates produced were subsequently used for bioethanol production [3]. A sequential fermentation process was proposed for a hydrogen production process with zero CO_2_ emission [4]. Cyanobacteria have been engineered to produce various bio-based chemicals from autotrophic CO_2_ assimilation [5–8]. On the other hand, PRK and Rubisco without the function of the full CBB cycle were shown to improve oil production in developing embryos of *Brassica napus* L [9]. This partial CBB cycle was also developed in Rubisco-dependent *Escherichia coli* (RDE) for the selection of Rubisco mutants that had better performance in terms of reaction rate and CO_2_/O_2_ selectivity [10–13]. The quantitative analysis of CO_2_ fixation in recombinant *E. coli* has been reported recently where 17 % of carbon was found to be directed to Rubisco-based engineered pathway in the presence of the carbonic anhydrase [14]. In our previous study, the feasibility of Rubisco-based engineered *E. coli* for in situ CO_2_ recycling during the fermentation of pentoses was demonstrated [15]. By enhancing the function of the homologous non-oxidative pentose phosphate pathway, Rubisco-based engineered *E. coli* can be further used to achieve a low CO_2_ emission during the fermentation of hexoses [16]. While Rubisco-based engineered pathway (that contains PrkA and Rubisco) is arguably compatible to the central metabolism of *E. coli* [16], it is perplexing to see the enhanced biomass accumulation and other physiological responses when form I Rubisco is heterologously expressed in *E. coli* [15]. It is also perplexing to see that only roughly 70 % of the carbon fed to the bacterial culture could be accounted for in the standard metabolic products. This low carbon recovery (the carbon recovery is defined as the mole of carbon of total fermentation products/the mole of carbon of consumed glucose) during fermentation occurred even though CO_2_ emission was effectively reduced by Rubisco-based engineered pathway [15, 16]. The low carbon recovery of 73 % can be seen for MZLFB + IP when pyruvate was not included for the calculation, see below. This unforeseen carbon distribution is also observed when Rubisco-based engineered pathway is heterologously expressed in *Saccharomyces cerevisiae* [17]. In this study, a recombinant *E. coli* strain with the deactivations of *ldh* and *frd* genes (encoding the lactate dehydrogenase and the fumarate reductase, respectively) was constructed to study the partition of carbon flow among the wild-type fermentation routes and Rubisco-based engineered pathway was quantified by examining the profiles of the end-metabolites. During which, several interesting phenotypes due to the overexpression of form I Rubisco were presented. Finally, the feasibility of mixotrophic *E. coli* as a platform for bio-based chemical productions was discussed.

## Results and discussion

### The pyruvate accumulation was found to be enhanced in *E. coli* in the presence of Rubisco

**Fig. 1:**
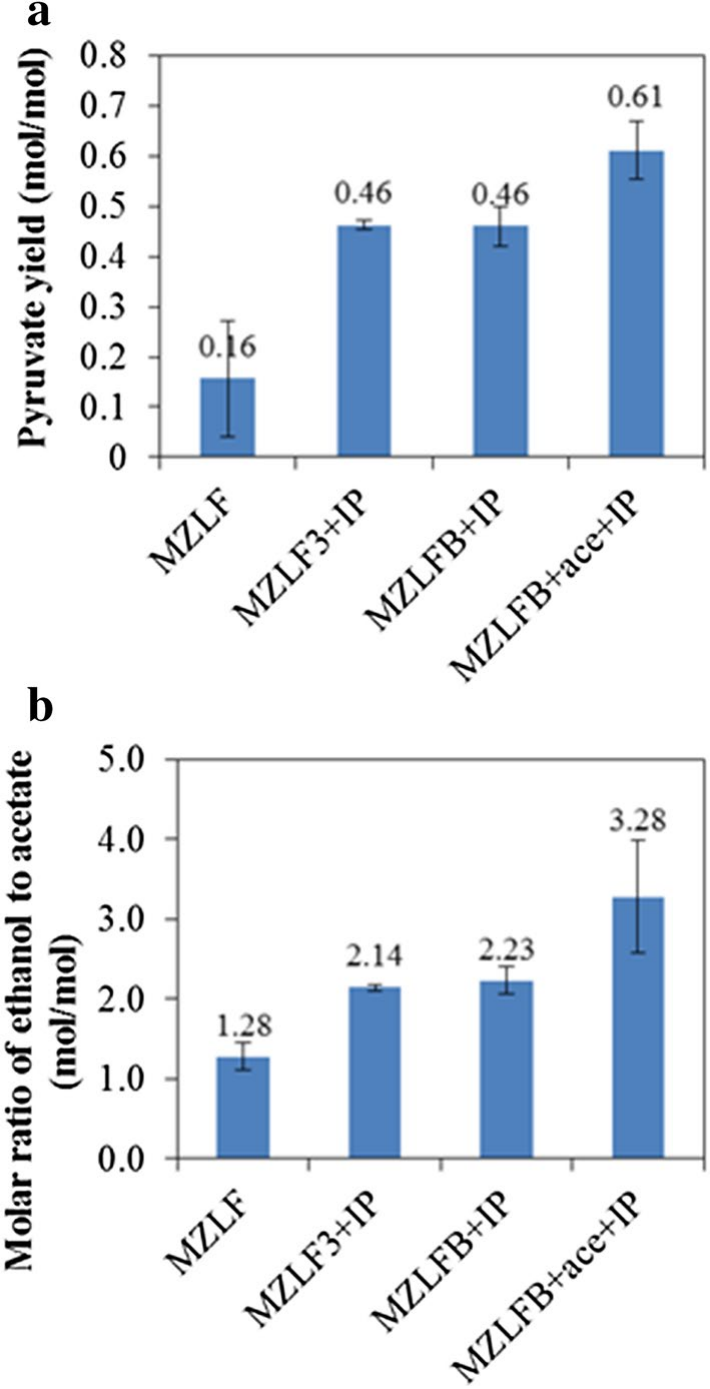
**a** Pyruvate yields and **b** molar ratios of ethanol to acetate of MZLF and its derived strains. The ace and IP represent the supplement of 2 g/l acetate and 0.02 mM IPTG at 8 h during the cultivation. *Error bars* represent standard deviations where independent data points are equal or greater than 6

It is consistently found from previous literature [15–17] that the carbon recovery generally decreased when Rubisco was heterologously expressed. This appears to diminish the usefulness of Rubisco for biotechnology applications. To investigate the carbon flow in *E. coli* strain containing Rubisco-based engineered pathway, *E. coli* strain MZLF, derived from *E. coli* strain MZ (*E. coli* BL21(DE3) Δ*zwf* [16]), was firstly constructed by deleting the non-lethal *ldh* and *frd* genes. Note that *zwf*, *ldh*, and *frd* genes encode glucose-6-phosphate 1-dehydrogenase, lactate dehydrogenase, and fumarate reductase, respectively. It can be seen in Fig. 1a that pyruvate was one of the major fermentation products for MZLF where the molar yield was 0.16, accounting for 9 % of the total carbon recovery (see data below). When the overexpression of Rubisco was achieved by the addition of isopropyl-β-d-1-thiogalacto-pyranoside (IPTG) during anaerobic culture of *E. coli* strains MZLF3 in M9 medium, a more than 2-fold increase in the yield of pyruvate can be observed. This unexpected result from MZLF3 was reproduced when MZLFB (MZLF containing *rbcLS* and *prkA*) was tested. The pyruvate yield can be increased by another 33 % by the addition of 30 mM acetate. Therefore, it can be concluded that it is Rubisco that induces the physiological response in *E. coli* MZLF and one of the main products is pyruvate. Note that an non-active Rubisco can also lead to the enhanced pyruvate production but not the CO_2_ assimilation, see below. This is consistent with our previous study that Rubisco alone can stimulate different physiological responses (enhanced biomass production and more) in *E. coli* as discussed in Introduction [15, 16]. It is also interesting to see in Fig. 1b that the overexpression of Rubisco led to a significant increase in the molar ratio of ethanol to acetate. While the molar ratio of ethanol to acetate for MZLF was 1.28, increases to 2.14 and 2.23 were respectively found for MZLF3 + IP and MZLFB + IP. A more than twofold increase from 1.34 to 3.28 was reported for MZLFB + ace + IP. Table 1 summarizes bacterial strains and plasmids used in this study. It should be noted that Rubisco used in MZLF3 or MZLFB is a mutant evolved from Rubisco of *Synechococcus* PCC6301 [13]. Nevertheless, we have shown that wild-type Rubisco also induced the pyruvate accumulation with a molar yield of 0.44 mol/mol-glucose (data not shown). Note that the elementary biomass composition of CH_1.77_O_0.49_N_0.24_ is used for the calculations of carbon recovery and molar yield [18].

**Table 1:** List of bacterial strains and plasmids used in this study

Name	Descriptions	Reference
*Bacterial strains*		
*E. coli* BL21 (DE3)	F-, *dcm*, *ompT*, *gal*, *lon*, *hsd*S_B_ (rB^−^, mB^−^), λ (DE3[*lac*I, *lac*UV5-T7 gene 1, *ind*1, *sam*7,*nin*5])	Lab stock
J3	*E. coli* BL21 (DE3) harboring *rbcLS*-pET30a + (M259T)	[15]
JB	*E. coli* BL21 (DE3) harboring both P_BAD_-his6-*prkA*-pACYC184 and *rbcLS*-pET30a + (M259T)	[15]
MZ	*E. coli* BL21 (DE3) Δ*zwf*	[16]
MZL	MZ, Δ*ldh*	This study
MZLF	MZL, Δ*frd*	This study
MZLF1	MZLF harboring P_BAD_-his6-*prkA*-pACYC184	This study
MZLF3	MZLF harboring *rbcLS*-pET30a + (M259T)	This study
MZLFB	MZLF harboring both P_BAD_-his6-*prkA*-pACYC184 and *rbcLS*-pET30a + (M259T)	This study
*Plasmids*		
pKD46	*araC*, *bla*, *oriR101*, *repA*101 (Ts), *araC*-*P*_*araB*_-*γ*-*β*-*exo* (encode λ Red recombinases), temperature-conditional replicon	[30]
pkD13	*bla*, FRT-*kan*-FRT	[30]
pCP20	*FLP* ^+^, λ *c*I857^+^,λ P_R_ Pep^ts^, *bla*, *catF*	[31]
P_BAD_-his6-*prkA*-pA CYC184	Recombinant plasmid carries *prkA* gene (derived from *Synechococcus* PCC7492) for the overexpresion of phosphoribulokinase (PrkA) under the control of P_BAD_	[13]
*rbcLS*-pET30a + (M259T)	Recombinant plasmid carries engineered *rbcLS* gene (originated from *Synechococcus* PCC6301) for the overexpresion of engineered Rubisco (M259T) under the control of P_T7_	[13]
*rbcLS*-pET30a + (M259T, K198G)	Recombinant plasmid carries engineered *rbcLS* gene (originated from *Synechococcus* PCC6301) for the overexpresion of engineered Rubisco (M259T and K198G) under the control of P_T7_	This study
*rbcLS*-pET30a + (M259T, K198G, D200G, E201G)	Recombinant plasmid carries engineered *rbcLS* gene (originated from *Synechococcus* PCC6301) for the overexpresion of engineered Rubisco (M259T, K198G, D200G, and E201G) under the control of PT7	This study
*rbcLS*-pET30a + (M259T, K198G, K172G)	Recombinant plasmid carries engineered *rbcLS* gene (originated from *Synechococcus* PCC6301) for the overexpresion of engineered Rubisco (M259T, K198G, and K172G) under the control of P_T7_	This study
*rbcLS*-pET30a + (M259T, K198G, K331G)	Recombinant plasmid carries engineered *rbcLS* gene (originated from *Synechococcus* PCC6301) for the overexpresion of engineered Rubisco (M259T, K198G, and K331G) under the control of P_T7_	This study

The distribution of C-2 fermentation products (i.e., ethanol and acetate) partly reflects the intracellular balances of ATP and the reducing equivalents. It has been demonstrated that the glycolytic flux is mainly controlled by the demand for ATP during the anaerobic growth of *E. coli* in the minimal medium [19, 20]. When the ATP demand, resulting from the anabolism, is strong and intense, the glycolic flux will accordingly increase to replenish ATP supply by the substrate-level phosphorylation. Moreover, additional ATP production can be achieved by the conversion of pyruvate derived from glycolysis. However, this route for the additional ATP production is restrained by surplus reducing equivalents generated from glycolysis where the conversion of pyruvate to ethanol is one of the main mechanisms to respire the reducing equivalents. The increase in the molar ratio of ethanol to acetate as shown in Fig. 1b infers that NADH may be produced in excess when Rubisco was overexpressed (see MZLF3 + IP). The introduction of PrkA in MZLFB (containing both Rubisco and PrkA), representing the extra demand for ATP, resulted in an additional increase of the ethanol:acetate ratio from 2.14 to 2.23 for MZLFB + IP. It is therefore suggested that the overproduction of NADH may result from an increase in glycolysis, which is because of the high demand for ATP in MZLF3 + IP and MZLFB + IP. These observations are consistent with the results when 30 mM of acetate were added, further increasing the molar ratio of ethanol to acetate from 2.23 to 3.28. The addition of acetate lowered the conversion of pyruvate to acetate by roughly 40 % (data not shown). The reduced acetate production resulted in reduced ATP production, thus further stimulating the glycolytic pathway to some extent. Note that the addition of 30 mM acetate during the cultivation of MZLF provided a pyruvate yield of 0.13 and an ethanol/acetate ratio of 1.25 (data not shown). This relatively same performance compared to MZLF as shown in Fig. 1 indicates that the enhanced pyruvate production observed for MZLFB + ace + IP (Fig. 1a) is because of the presence of Rubisco. And the enhanced pyruvate production can be attributed to the unbalanced ATP production. The enhanced glycolytic activity accompanied by enhanced bacterial growth is consistent with our previous study when Rubisco was aerobically overexpressed in *E. coli* BL21 (DE3) [15].

Pyruvate is not a typical fermentation product of anaerobic cultivation of *E. coli*. Instead, it is an important metabolic node that is associated with the metabolisms of lactate, acetate, ethanol, and succinate [21]. It is also involved in the anaplerosis (through phosphoenolpyruvate-pyruvate-oxaloacetate) as well as glucose metabolism (through phosphotransferase system, PTS) [22]. The enhanced glycolysis due to the overexpression of Rubisco should lead to vigorous function of PTS where PEP is converted to pyruvate. At the same time, the enhanced anaplerotic metabolism has been shown to occur when Rubisco is aerobically overexpressed in *E. coli* BL21 (DE3) [15] or anaerobically in *E. coli* MZ [16] and MZLFB (see results below). The enhanced anaplerosis competes with PTS for PEP. This may further intensify the activity of glycolysis and PTS and thus results in the enhanced pyruvate production.

A second pathway for the enhanced pyruvate accumulation by the presence of Rubisco can be argued as follows. Pyruvate should be primarily directed to the production of C-2 fermentation products since *ldh* and *frd* have been deactivated in *E. coli* MZLF. The anaerobic conversion of pyruvate to C-2 fermentation products is started by the enzymatic reaction catalyzed by pyruvate formate-lyase (PFL), where the reaction products are acetyl-CoA and formate. The enhanced pyruvate regulation may result from the down-regulation of the transcription of the *focA*-*pflB* operon and thus decrease the total activity of PFL. The regulation of gene transcriptions involves a complex network of global and local regulators. In *E. coli*, the transcription of about one-half of genes is regulated by seven global regulators including ArcA, Crp, Fis, Fnr, Ihf, Lrp, and NarL [23]. It is known that the transcription of the *focA*-*pflB* operon is positively regulated by the redox-dependent transcriptional activators of FNR and ArcA [24, 25] whereas, ArcA is found to be critical to the transcription of the *focA*-*pflB* operon [25]. It is surprising to see that the transcriptional level of *arcA* (encodes the aerobic respiration control protein) is down-regulated in *E. coli* strains J3 and JB due to the presence of Rubisco, as noted in Table 2. This should also hold true when Rubisco is expressed in MZLF. Several studies have shown that the disruption of *arcA* in *E. coli* would activate the glyoxylate shunt [26, 27]. This is consistent with NGS data where the transcription levels of genes associated with the glyoxylate shunt were indeed enhanced, as shown in Additional file 1. The *arcA* mutant strain also exhibited a decrease in the acetate yield and an increase in biomass yield [26, 27]. These phenotypes of *arcA* mutant strain are consistent with our data as discussed here and below. Therefore, the down-regulation of *arcA* transcription provides a phenomenological explanation for the results of pyruvate accumulation where the transcription of the *focA*-*pflB* operon may decrease. More work is needed to elucidate the detailed mechanism of Rubisco-induced phenotypes.

**Table 2:** Transcriptional levels of global regulators in Rubisco-based *E. coli*

Global regulator	log_2_Ratio in J3	log_2_Ratio in JB
*arcA*	−0.81	−0.61

The definition of log_2_Ratio can be found in Text S1. All log_2_Ratio reported here are statistically significant where all p-values are less than 1 × 10^−3^ and all False Discovery Rates (FDRs) are less than 0.05

### Carbon dioxide is recycled and converted into fermentation products and biomass by Rubisco-based engineered pathway

**Fig. 2:**
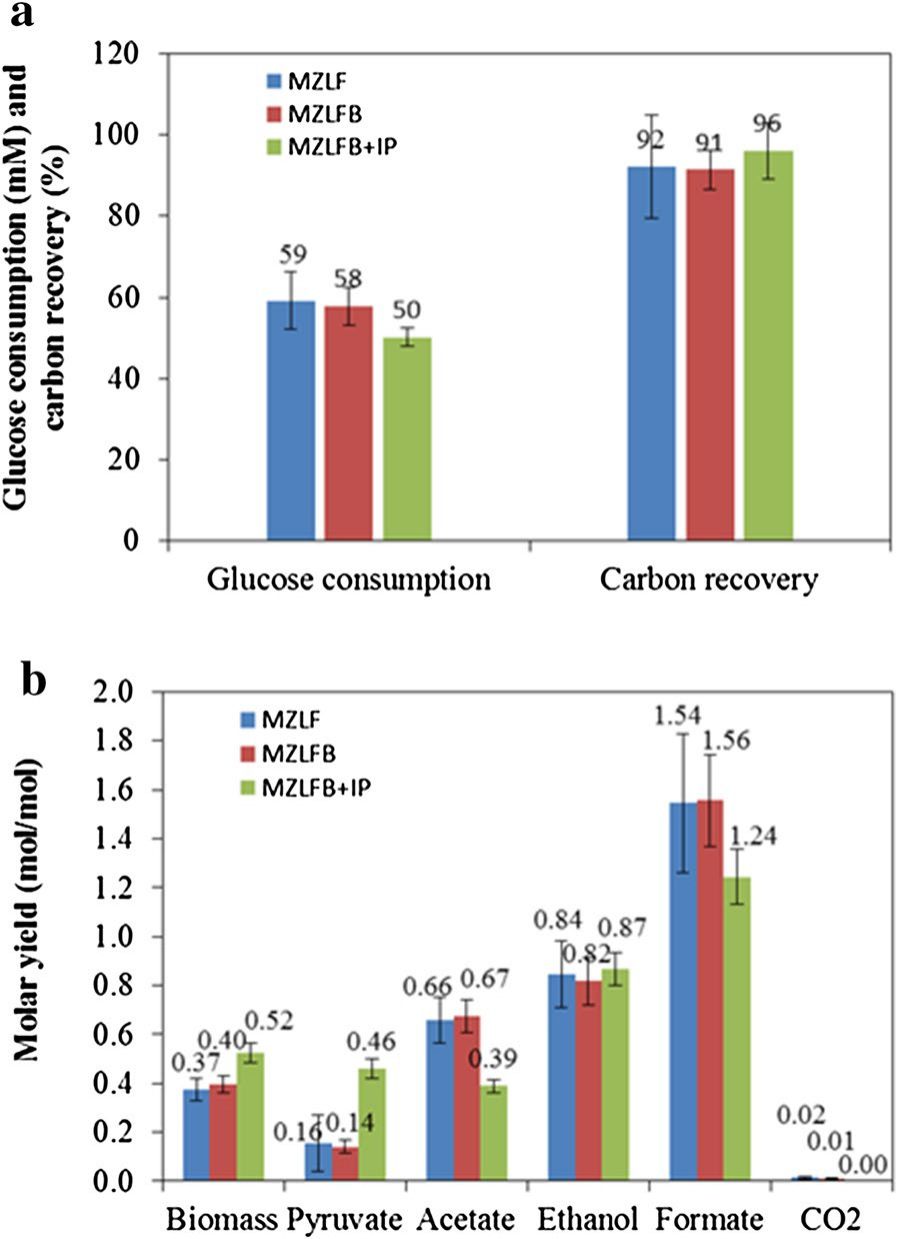
**a** Glucose consumption, carbon recovery, and **b** fermentation product yield of MZLF and its derived strains. The IP represents the supplement of 0.02 mM IPTG at 8 h during the cultivation. *Error bars* represent standard deviations where independent data points are equal or greater than 6. The elementary biomass composition of CH_1.77_O_0.49_N_0.24_ is used for the calculations of carbon recovery and molar yield

With the elucidation of the Rubisco-induced responses, it is interesting to verify the carbon recovery and carbon distribution for *E. coli* containing Rubisco-based engineered pathway. It can be seen in Fig. 2a that while the carbon recovery for MZLF is 92 %, the carbon recovery for MZLFB and MZLFB + IP is 91 and 96 %, respectively. By including the contribution of pyruvate accumulation, the perplexing problem of low carbon recovery (ca. 70 %) during fermentation by *E. coli* containing Rubisco-based engineered pathway has been solved [15, 16]. Moreover, two important analyses can be made with good carbon recoveries. First, it is interesting to see a significant difference in the carbon distribution between strains MZLF and MZLFB + IP (Fig. 2b). In addition to the increase in pyruvate production, the molar yield of biomass increased from 0.37 to 0.52, and it is therefore suggested that Rubisco may be a key to stimulate bacterial growth. Second, it can be observed from Fig. 2b that the amount of carbons needed to match the increase in carbon yields of the biomass and pyruvate cannot be simply balanced by the decrease in the carbon yield of acetate alone. However, when C-1 compounds are taken into consideration, a good carbon balance during the carbon re-distribution between MZLF and MZLFB + IP can be made. Therefore, it is suggested that CO_2_, derived from formate, will also be converted to the biomass and fermentation products in MZLFB + IP.

Considering the profile of fermentation products where C-3, C-2, and C-1 account for 83 and 86 % of total end metabolites for MZLFB and MZLFB + IP, respectively (Table 3), a reasonable conclusion is that glycolysis (Eq. (1), below) and subsequent C-2 fermentation routes (Eqs. 2 and 3, below) are the major metabolic fate for glucose. The 83 and 86 % of C-1, C-2, and C-3 metabolites are calculated from the data in Table 3 by taking pyruvate, acetate, ethanol, formate, and CO2 into considerations for the calculation of the carbon recovery, whereas the lactate yield is small enough to be neglected. Two additional routes are appended to account for the activity of Rubisco-based engineered pathway (Eqs. 4 and 5, below). Since each equation has distinct stoichiometry for C-1 metabolism, where glycolysis produces zero C-1 (Eq. 1), conventional C-2 fermentation produces two C-1 (Eqs. 2 and 3), and the Rubisco-based engineered pathway produces 1.2 C-1 (Eqs. 4 and 5), the distribution of glucose flux among metabolic fates (Eqs. 1–5) for MZLFB can be simply determined by the profile of the end metabolite. In other words, the final concentrations of C-3, C-2, and C-1 metabolites are interdependent. Detailed derivations for Eqs. 1–5 are presented in Additional file 1.

1 $${\text{glucose }} + 2 {\text{ADP}} + 2 {\text{NAD}}^{ + } \to 2 {\text{pyruvate}} + 2 {\text{ATP}} + 2 {\text{NADH}}$$

2 $${\text{glucose}} + 2 {\text{ADP}} + 2 {\text{NADH}} \to 2 {\text{ethanol}} + 2 {\text{formate}} + 2 {\text{ATP}} + 2 {\text{NAD}}^{ + }$$

3 $${\text{glucose }} + 4 {\text{ADP}} + 2 {\text{NAD}}^{ + } \to 2 {\text{acetate}} + 2 {\text{formate}} + 4 {\text{ATP}} + 2 {\text{NADH}}$$

4 $${\text{glucose }} + 4.8 {\text{NADH}} \to 2.4 {\text{ethanol}} + 1.2 {\text{formate}} + 4.8 {\text{NAD}}^{ + }$$

5 $${\text{glucose }} + 2.4 {\text{ADP}} \to 2.4 {\text{acetate}} + 1.2 {\text{formate}} + 2.4 {\text{ATP}}$$

**Table 3:** Fermentation profiles of *E. coli* MZLF derived strains

*E. coli* strains	Glucose consumption	Molar yield (mmol/mmol)								Carbon recovery (%)
	(mM)	Pyr	Ace	EtOH	Lac	Suc	For	CO_2_	Biomass	
MZLF	59 ± 7	0.16 ± 0.12	0.66 ± 0.09	0.84 ± 0.14	0.04 ± 0.02	0	1.54 ± 0.28	0.02 ± 0.005	0.37 ± 0.05	92 ± 9
MZLF3 + IP	55 ± 1	0.46 ± 0.02	0.41 ± 0.00	0.87 ± 0.01	0.03 ± 0.00	0	1.30 ± 0.02	0.001 ± 0.00	0.54 ± 0.02	98 ± 1
MZLFB	58 ± 5	0.14 ± 0.03	0.67 ± 0.07	0.82 ± 0.10	0.04 ± 0.01	0	1.56 ± 0.19	0.01 ± 0.003	0.40 ± 0.03	92 ± 5
MZLFB + IP	50 ± 2	0.46 ± 0.04	0.40 ± 0.03	0.87 ± 0.07	0.03 ± 0.01	0	1.24 ± 0.11	0	0.52 ± 0.04	96 ± 7
MZLFB + ace + IP	51 ± 1	0.61 ± 0.06	0.25 ± 0.05	0.82 ± 0.04	0.02 ± 0.0	0	1.13 ± 0.06	0	0.50 ± 0.03	94 ± 4

The calculation of carbon recovery includes ethanol, acetate, formate, lactate, succinate, malate, CO_2_, and biomass. The elementary biomass composition of CH_1.77_O_0.49_N_0.24_ is used for the calculations of carbon recovery [18]

IP and ace represent the supplement of 0.05 mM IPTG and 30 mM acetate at 8 h during the cultivation

*Pyr* pyruvate, *Ace* acetate, *EtOH* ethanol, *Lac* lactate, *Suc* succinate, *For* formate

**Fig. 3:**
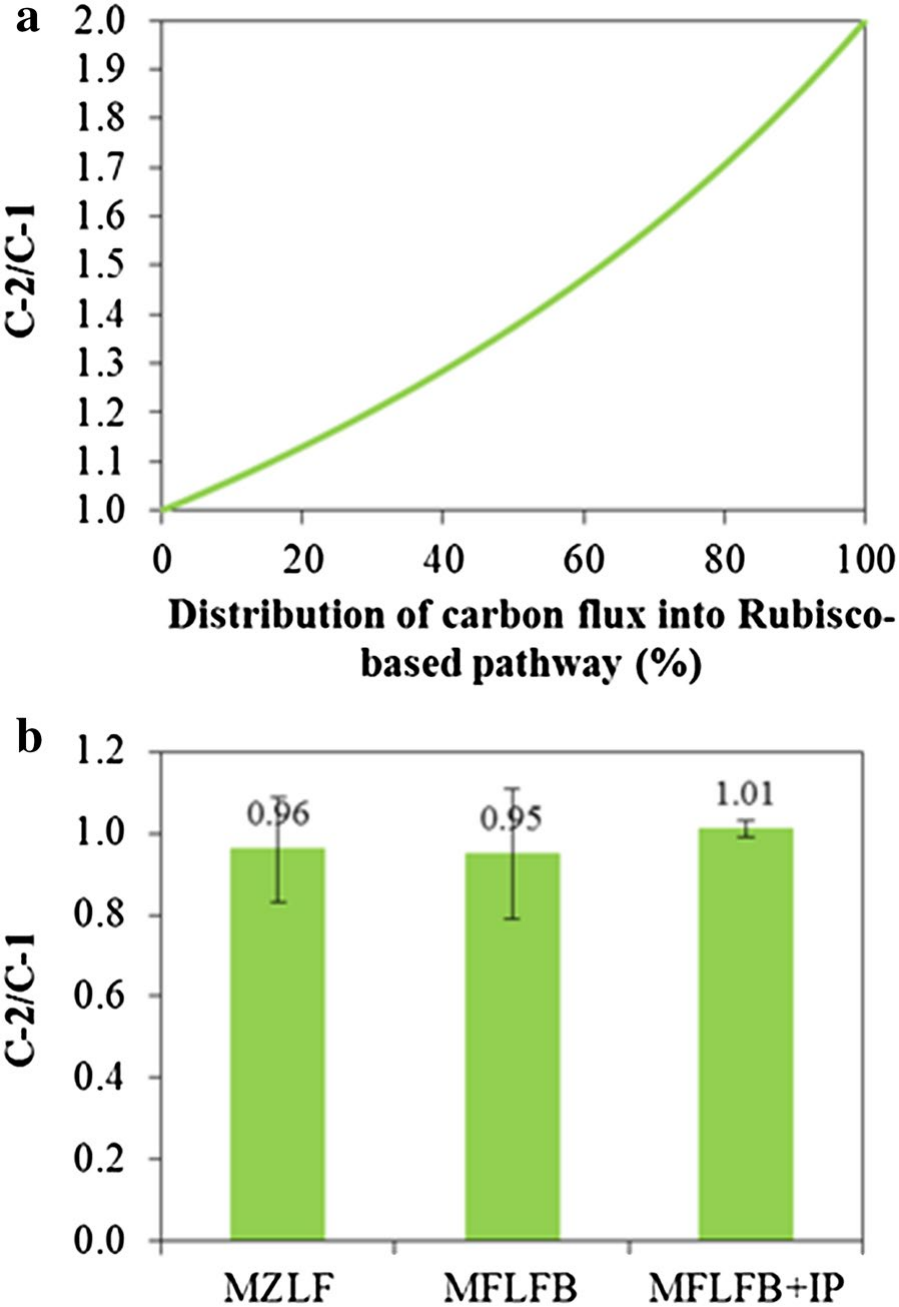
**a** The theoretical calculation of C-2/C-1 ratio based on the distribution of carbon flux into Rubisco-based engineered pathway. **b** The experimental data of the C-2/C-1 ratio for *E. coli* MZLF and its derived strains. C-2 represents the sum of ethanol and acetate. C-1 represents the sum of formate and CO_2_. IP represents the supplement of 0.02 mM IPTG at 8 h during the cultivation. *Error bars* represent standard deviations where independent data points are equal or greater than 6

According to Eqs. 2–5, the theoretical C-2/C-1 ratio can be calculated as a function of the distribution of glucose flux between conventional fermentation route and Rubisco-based engineered pathway (Fig. 3a). When the C-2/C-1 ratio is 1, glucose flux to Rubisco-based engineered pathway should be 0 %. This scenario represents a conventional fermentation process as shown in Eqs. 2 and 3. The C-2/C-1 ratio non-linearly increases from 1 to 2 as the contribution of the Rubisco-based engineered pathway described by Eqs. 4 and 5 increases from 0 to 100 %. It can be seen in Fig. 3b that the experimental data showed that C-2/C-1 for MZLF was 0.96. With the addition of 0.02 mM IPTG to increase the expression of Rubisco in MZLFB, the molar ratio of C-2/C-1 increased to 1.01. This indicates that in addition to conventional C-2 fermentation routes (Eqs. 2 and 3), Rubisco-based engineered pathway should participate in C-2 fermentative production as given by Eqs. 4 and 5. Furthermore, the 5 % increase in the molar ratio of C-2/C-1 for MZLFB + IP corresponds with roughly 9 % of the glucose flux flew into Rubisco-based engineered pathway. This corresponds to C-2/C-1 of 1.05 which is a 5 % increase compared to the base whose C-2/C-1 is 1.00. The deviation of C-2/C-1 for MZLF from the theoretical calculation can be attributed to the fact that CO_2_ evolved from the production of biomass is included in the calculation of C-2/C-1. The percentage of the carbon flux directing into Rubisco-based engineered pathway is therefore under-estimated. Note that it was proposed that 2.8 mol of CO_2_ would be evolved when one mole of biomass was produced [26].

**Fig. 4:**
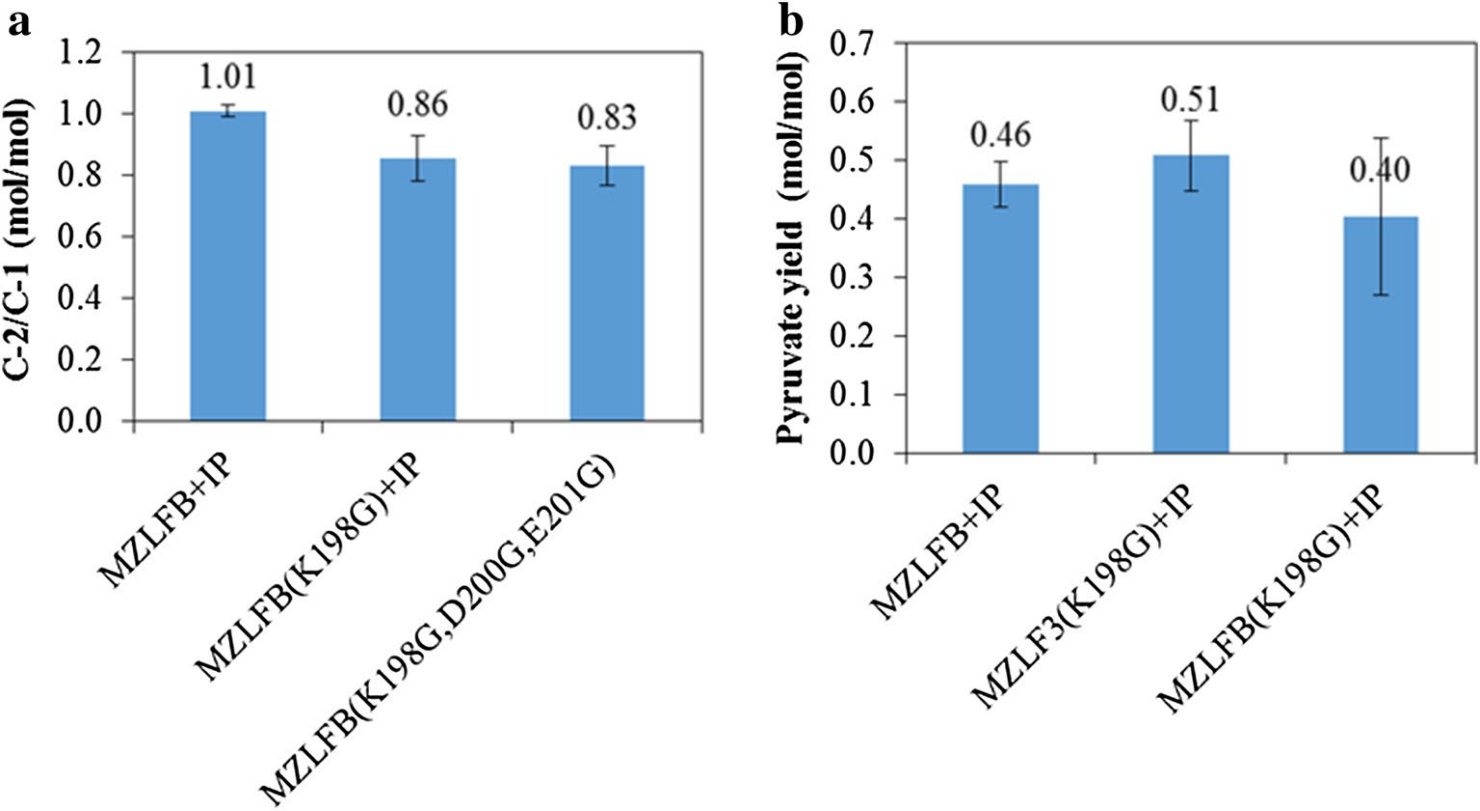
**a** C-2/C-1 ratios of MZLF, MZLFB, MZLFB (K198G), and MZLFB (K198G,K172G). **b** Pyruvate yields of MZLFB + IP, MZLF3 (K198G) + IP, and MZLFB (K198G) + IP. *Error bars* represent standard deviations where independent data points are equal or greater than 3

To verify the in vivo activity of Rubisco-based engineered pathway, two Rubisco mutants were prepared to replace the parental Rubisco in MZLFB. The first Rubisco mutant was Rubisco (K198G) where the lysine at the position 198 is the essential binding site for CO_2_ [28]. The second Rubisco mutant was prepared by introducing additional amino acid substitution to get Rubisco (K198G, K172G). The lysine at the position 172 is associated with the binding of ribulose-1,5-bisphosphate [28]. While the molar ratio of C-2/C-1 for MZLF was 0.96, the increase in the C-2/C-1 ratio to 1.01 for MZLFB + IP demonstrated the proper function of Rubisco-based engineered pathway. When Rubisco(K198G) was substituted for parental Rubisco, a dramatic decrease in the C-2/C-1 ratio to 0.86 was observed (Fig. 4a). It can be seen that Rubisco with double mutations of K198G and K172G had the C-2/C-1 ratio of 0.83, which is also far below the performance of MZLFB + IP. This control experiment demonstrates the in vivo activity of Rubisco-based engineered pathway. Thus, the Rubisco-based engineered pathway can be deactivated by mutating lysine at position 198 of the primary structure. Note that the overexpression of Rubisco (K198G) can be confirmed by SDS-PAGE as shown in Additional file 1. Also noted was that the overexpression of Rubisco (K198G) also enhanced the accumulation of pyruvate (Fig. 4b). This indicates that the enhanced pyruvate accumulation is not caused by the carboxylation reaction catalyzed by Rubisco.

### The feasibility of mixotrophic *E. coli* as a platform for bio-based chemical productions

**Fig. 5:**
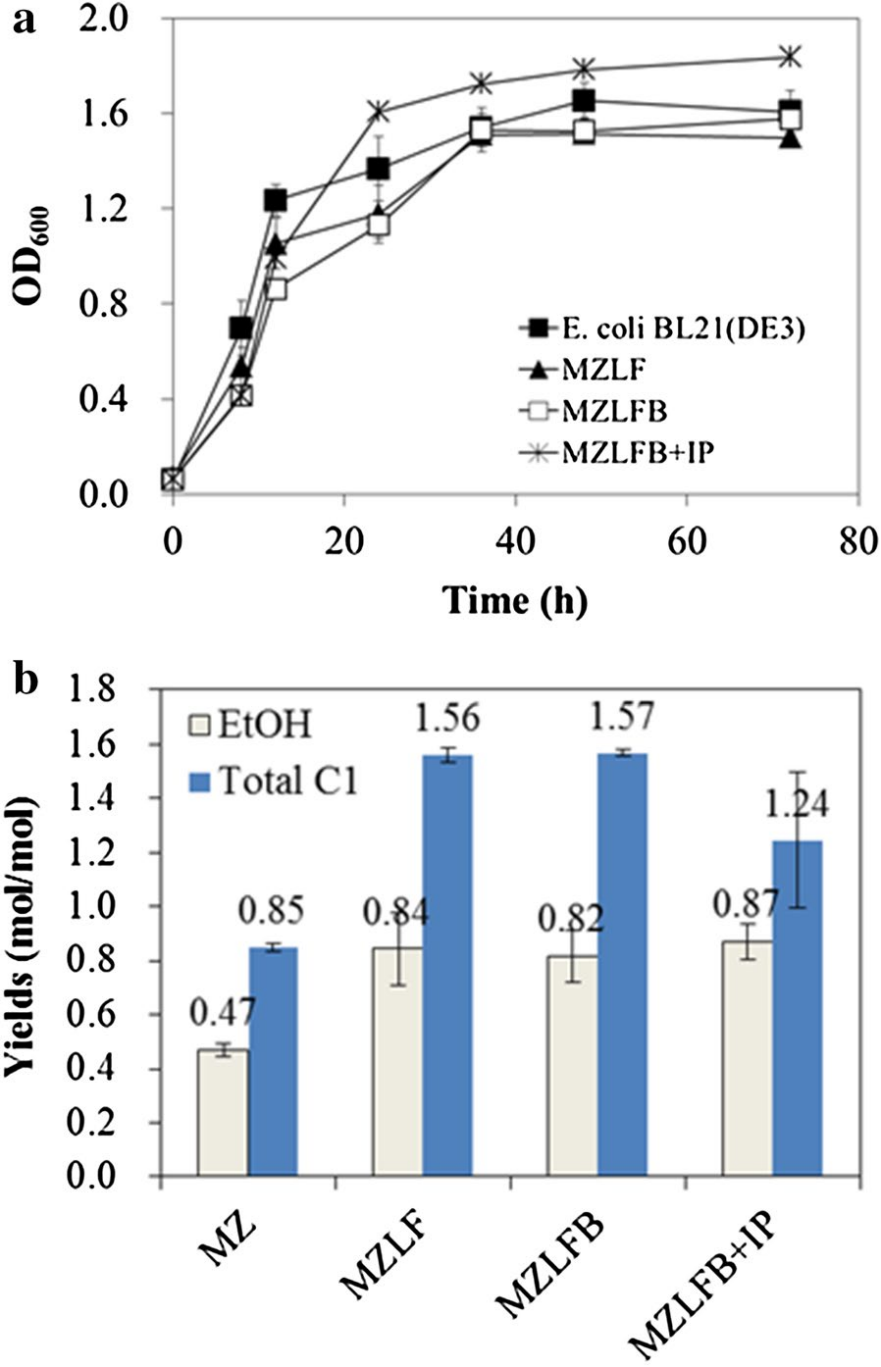
**a** Bacterial growth curves of *E. coli* BL21 (DE3), MZLF, MZLFB, and MZLFB + IP. IP represents the supplement of 0.02 mM IPTG at 8 h during the cultivation. **b** Ethanol and total C-1 yields of MZ and its derived strains. *Error bars* represent standard deviations where independent data points are 6

The feasibility of mixotrophic *E. coli* has been demonstrated (see Fig. 5a). It is well known that the expression of PrkA in *E. coli* is a burden for bacterial growth yet the co-expression of Rubisco can alleviate such retarded growth [13, 15, 16]. These previous studies can be illuminated not only with respect to carbon flow but also to the intracellular energy balance. It can be seen that in a regime where 9 % of the carbon is directed into Rubisco-based engineered pathway, the energy can still be self-balancing while CO_2_ is recycled. In such case, the growths of MZLFB and MZLB + IP were comparable or even better than that of wild-type *E. coli*. This is consistent with our previous suggestion of the compatibility of Rubisco-based engineered pathway with the central metabolism of *E. coli* [16]. Moreover, there is plenty of formate (1.24 mol/mole glucose) after directing 9 % of the carbon to Rubisco-based engineered pathway, indicating that energy may not be a constraint for the further forcing of even more carbon into Rubisco based engineered pathway.

The feasibility of mixotrophic *E. coli* can also be perceived by examining ethanol production and the associated C-1 accumulation. It can be seen in Fig. 5b that *E. coli* strain MZ produced 0.47 mol-ethanol/mol-glucose with a 0.85 mol-C-1/mol-glucose. By de-activating *ldh* and *frd* genes from MZ, *E. coli* strain MZLF produced 0.84 mol-ethanol/mol-glucose (79 % increase) while C-1 production was proportionally increased to 1.56 mol-C-1/mol-glucose. The introduction of Rubisco-based engineered pathway into MZLF enhanced the ethanol production by 4 % while decreasing total C-1 production by 21 %. This is in agreement with the theoretical calculation that 9 % of carbon flux was directed into Rubisco-based engineered pathway. Note that some recycled C-1 could arguably become biomass since the biomass yield increase from 0.37 for MZLF to 0.52 for MZLFB + IP.

## Conclusions

Here, we report for the first time the complete profile of fermentation products using *E. coli* MZLF and its derived strains. The profiles reveal that the heterologous expression of form I Rubisco has a strong impact on the central metabolism of a non-phototrophic heterotroph *E. coli*, resulting in significant increases in the production of biomass and pyruvate. By examining the comprehensive profile of fermentation products, it is estimated that 9 % of glucose consumption is directed from glycolysis to Rubisco-based engineered pathway. The co-existence of glycolysis and Rubisco-based engineered pathway in *E. coli* MZLFB poses an example as the mixotroph where the principle characteristics of MZLFB are the high bacterial growth and the low CO_2_ emission.

## Methods

### Bacterial strains and plasmids

All strains and plasmids used in this study are listed in Table 1. The *ldh* gene knock-out mutant derived from *E. coli* strain MZ and the *frd* gene knock-out mutant derived from *E. coli* strain MZL were constructed through the one-step inactivation procedure [29, 30]. The sequences of primers used for the synthesis of linear DNA fragments for Red-mediated recombination can be found in Additional file 1. More information regarding the construction of mutant strains can be found in [16].

The recombinant plasmid *rbcLS*-pET30a + (M259T,K198G) was derived from *rbcLS*-pET30a + (M259T) by the Q5 Site-Directed Mutagenesis Kit (New England BioLabs^®^ Inc., the USA), where primers rbcL-K198G-F and rbcL-K198G-R were used, see Additional file 1. *rbcLS*-pET30a + (M259T,K198G,D200G,E201G), *rbcLS*-pET30a + (M259T,K198G,K172G), and *rbcLS*-pET30a + (M259T,K198G,K331G) were generated subsequently from *rbcLS*-pET30a + (M259T,K198G). Four recombinant plasmids were subject to DNA sequencing (serviced by Biotechnology center at National Chung Hsing University, Taiwan). All primers used for the amplification of mutation were listed in (Additional file 1).

### Culture media and growth conditions

*Escherichia coli* strains used for fermentation study were grown anaerobically at 200 rpm and 37 °C in fresh 25-ml M9 defined medium containing (per liter): 12.8 g Na_2_HPO_4_·7 H_2_O; 3 g KH_2_PO_4_; 0.5 g NaCl; 1.0 g NH_4_Cl; 0.24 g MgSO_4_; 0.011 g CaCl_2_; and 20 g glucose. An anaerobic culture environment was achieved in an sealed serum bottle as described previously [31]. Initial OD_600_ was adjusted to 0.05. The pH was adjusted to 8 at the fermentation times of 0, 8, and 24 h. The respective concentrations of streptomycin, chloramphenicol and kanamycin used were 50, 34, and 50 μg/ml. The isopropyl-β-d-1-thiogalactopyranoside (IPTG) and acetate was added at 8 h when needed.

### Analytical methods

The cell density was measured at 600 nm using a UV–Vis spectrophotometer (GENESYS 10S, Thermo Scientific, USA). The gaseous CO_2_ concentration in the headspace of the cultures was measured by a diffusive infrared-based CO_2_ analyzer (Sentry ST303). The total CO_2_ concentration was calculated based on the gaseous CO_2_ concentration and the detailed calculation has been described in [15, 16].

Samples for quantification of residual glucose or extracellular metabolites were collected from the culture solutions followed by the centrifugation for 5 min at 17,000×*g* to remove cell pellets. The supernatant was filtered by a 0.2 μm PVDF filter before sample injection. The concentration of residual glucose was determined by either HPLC or DNS methods [32]. Characterization and quantification of extra-cellular formate, acetate, ethanol, lactate, succinate, and pyruvate were performed by Thermo ScientificTM DionexTM Ulitmate 3000 LC Systems. The separation of the mixture was achieved with the HPLC column Thermo scientific HyperREZ XP Carbohydrate H^+^ (300 mm × 7.7 mm 8 μm) where the measurement was done with a refractive index (RI) detector. The mobile phase was 5 mM H_2_SO_4_. The temperature was maintained at 65 °C while the flow rate was maintained at 1.0 ml per minute. The sample injection was done by an autosampler whereas the injection volume is 20 μl.

The qualitative and quantitative analysis of pyruvate was further confirmed by the use of Pyruvate Colorimetric/Fluorometric Assay Kit (Biovision Inc., the USA). Basically, 50 µl proprietary Reaction Mix, containing 46 µl of Assay Buffer, 2 µl of Pyruvate Probe, and 2 µl Enzyme Mix, were mixed with 50 µl of sample. Incubate the mixtures in a light-free condition for 30 min at room temperature. The absorbance of samples at 570 nm was measured using a microplate reader.

### Quantification of mRNA expression level

The RNA sequencing was done by Genomics BioSci & Tech Ltd and used to quantify the expression level of mRNA. Primary sequencing data of cDNA library were performed by Illumina HiSeq™ 2000 and the data mapping was done with SOAPaligner/SOAP2. *E. coli* strains J3 together with *E. coli* BL21 (DE3) as the control experiment were aerobic cultivated in LB medium supplemented with 20 g/l L-arabinose [15]. Meanwhile, *E. coli* strains JB together with *E. coli* BL21 (DE3) as the control experiment were anaerobic cultivated in M9 medium supplemented with 20 g/l d-glucose as presented above. The samples for J3 transcriptome analysis were collected at 24 h while the samples for JB transcriptome analysis were collected at 24 h. Four samples for RNA-seq were shipped out with dry ice bath. Detailed protocol for sample preparation, RNA-seq, and data analysis can be found in Text S1 and [16].

## Additional files

10.1186/s12934-016-0530-7 Supplemental Materials and Methods.

## Abbreviations

The CBB cycle: The Calvin–Benson–Bassham cycle
PRK: phosphoribulokinase
PrkA: phosphoribulokinase originated from *Synechococcus* PCC7492
Rubisco: Ribulose-1,5-bisphosphate carboxylase/oxygenase
RDE: Rubisco-dependent *Escherichia coli*
C-1 compounds: represent formate and CO_2_
C-2 compounds: represent ethanol and acetate
PFL: pyruvate formate-lyase
ArcA: aerobic respiration control protein
SDS-PAGE: sodium dodecyl sulfate–polyacrylamide gel electrophoresis
IPTG: isopropyl-β-d-1-thiogalactopyranoside
