# Supplementary material for: The comprehensive profile of fermentation products during in situ CO2 recycling by Rubisco-based engineered Escherichia coli
Source: Microb Cell Fact. 2016 Aug 2;15:133. doi: 10.1186/s12934-016-0530-7 (PMC4971712; doi:10.1186/s12934-016-0530-7)
Supplement: Supplementary file 1 — 10.1186/s12934-016-0530-7 Supplemental Materials and Methods. [file 12934_2016_530_MOESM1_ESM.pdf]

Text S1. Supplemental Materials and Methods.

Contents of Text S1

a. Quantification of mRNA expression level
b. Derivation of stoichiometric models for glucose metabolism in *E. coli*

a. Quantification of mRNA expression level

The expression level of mRNA of a bacterial culture can be quantified by reads per kilobase transcriptome per million mapped reads (RPKM) defined as follows (1):

$$\mathrm{RPKM} = \frac{10^6 C}{NL/10^3}$$

where C is the reads of the target gene, N is the reads of total genes, and L is the number of bases of the target gene. To accommodate the deviation of RPKM for J3 and JB caused by the heterologous expression of Rubisco and/or PrkA, a modified RPKM (M-RPKM) is defined for strain j:

$$\mathrm{M\text{-}RPKM} = \frac{10^6 C}{N'L/10^3}$$

where N' is calculated by subtracting the reads of *rbcLS* and/or *prkA* from the reads of total genes.

The differential expression of mRNA between wild-type *E. coli* BL21(DE3) and strain j is now as follows:

$$\log_2\mathrm{Ratio} = \frac{\mathrm{M\text{-}RPKM}_{\mathrm{j,x}}}{\mathrm{RPKM}_{\mathrm{BL21(DE3),x}}}$$

where $\mathrm{M\text{-}RPKM}_{\mathrm{j,x}}$ is the modified RPKM of gene x for strain j where $\mathrm{RPKM}_{\mathrm{BL21(DE3),x}}$ is the RPKM of gene x for wild-type *E. coli* BL21(DE3). It is suggested that a statistically significant $\log_2$Ratio should be accompanied by a p-value of less than 1×10^{-3} and a FDR of less than 0.05 (2).

b. Derivation of stoichiometric models for glucose metabolism in *E. coli.*

(a) $\text{glucose} + 2\ \text{ADP} + 2\ \text{NADH} \rightarrow 2\ \text{ethanol} + 2\ \text{formate} + 2\ \text{ATP} + 2\ \text{NAD}^+$

(b) $\text{glucose} + 4\ \text{ADP} + 2\ \text{NAD}^+ \rightarrow 2\ \text{acetate} + 2\ \text{formate} + 4\ \text{ATP} + 2\ \text{NADH}$

(c) $\text{glucose} + 4.8\ \text{NADH} \rightarrow 2.4\ \text{ethanol} + 1.2\ \text{formate} + 4.8\ \text{NAD}^+$

(d) $\text{glucose} + 2.4\ \text{ADP} \rightarrow 2.4\ \text{acetate} + 1.2\ \text{formate} + 2.4\ \text{ATP}$

(a) $\mathbf{glucose + 2\ ADP + 2\ NADH \rightarrow 2\ ethanol + 2\ formate + 2\ ATP + 2\ NAD^+}$

① $\text{glucose} + 2\ \text{ADP} + 2\ \text{NAD}^+ \xrightarrow{\text{Glycolysis}} 2\ \text{pyruvate} + 2\ \text{ATP} + 2\ \text{NADH}$

② $\text{pyruvate} + \text{CoA} \xrightarrow{pflB} \text{acetyl-CoA} + \text{formate}$

③ $\text{acetyl-CoA} + 2\ \text{NADH} \xrightarrow{adhE} \text{ethanol} + 2\ \text{NAD}^+ + \text{CoA}$

(a) can be derived from ①+②×2+③×2

(b) $\mathbf{glucose\ +4\ ADP+2\ NAD^{+}\rightarrow 2\ acetate+2\ formate+4\ ATP+2\ NADH}$

① $\text{glucose} + 2\ \text{ADP} + 2\ \text{NAD}^{+} \xrightarrow{\text{Glycolysis}} 2\ \text{pyruvate} + 2\ \text{ATP} + 2\ \text{NADH}$

② $\text{pyruvate} + \text{CoA} \xrightarrow{pflB} \text{acetyl-CoA} + \text{formate}$

④ $\text{acetyl-CoA} \xrightarrow{pta} \text{acetyl-P} + \text{CoA}$

⑤ $\text{acetyl-P} + \text{ADP} \xrightarrow{ackA} \text{acetate} + \text{ATP}$

(b) can be derived from ①+②×2+④×2+⑤×2

(c) $\mathbf{glucose\ +4.8\ NADH\rightarrow 2.4\ ethanol+1.2\ formate+4.8\ NAD^{+}}$

⑨ $5\ \text{glucose} + 6\ \text{ATP} \xrightarrow[+rpe\ \text{and}\ rpi]{\text{non-oxidative pentose phosphate pathway}} 6\ \text{ribulose-5-phosphate} + 6\ \text{ADP}$

⑩ $\text{ribulose-5-phosphate} + \text{ATP} \xrightarrow{prkA} \text{ribulose-1,5-bisphosphate} + \text{ADP}$

⑪ $\text{ribulose-1,5-bisphosphate} + CO_2 \xrightarrow{rbcLS} 2\ \text{3-phosphoglycerate}$

⑫ $\text{3-phosphoglycerate} + \text{ADP} \xrightarrow{gpm+eno+pyk} \text{pyruvate} + \text{ATP}$

② $\text{pyruvate} + \text{CoA} \xrightarrow{pflB} \text{acetyl-CoA} + \text{formate}$

③ $\text{acetyl-CoA} + 2\ \text{NADH} \xrightarrow{adhE} \text{ethanol} + 2\ \text{NAD}^{+} + \text{CoA}$

(c) can be derived from ⑨×0.2+(⑩+⑪)×1.2+(⑫+②+③)×2.4

(d) $\mathbf{glucose\ +2.4\ ADP\rightarrow 2.4\ acetate+1.2\ formate+2.4\ ATP}$

⑨ $5\ \text{glucose} + 6\ \text{ATP} \xrightarrow[+rpe\ \text{and}\ rpi]{\text{non-oxidative pentose phosphate pathway}} 6\ \text{ribulose-5-phosphate} + 6\ \text{ADP}$

⑩ $\text{ribulose-5-phosphate} + \text{ATP} \xrightarrow{prkA} \text{ribulose-1,5-bisphosphate} + \text{ADP}$

⑪ $\text{ribulose-1,5-bisphosphate} + CO_2 \xrightarrow{rbcLS} 2\ \text{3-phosphoglycerate}$

⑫ $\text{3-phosphoglycerate} + \text{ADP} \xrightarrow{gpm+eno+pyk} \text{pyruvate} + \text{ATP}$

② $\text{pyruvate} + \text{CoA} \xrightarrow{pflB} \text{acetyl-CoA} + \text{formate}$

④ $\text{acetyl-CoA} \xrightarrow{pta} \text{acetyl-P} + \text{CoA}$

⑤ $\text{acetyl-P} + \text{ADP} \xrightarrow{ackA} \text{acetate} + \text{ATP}$

(d) can be derived from ⑨×0.2+(⑩+⑪)×1.2+(⑫+②+④+⑤)×2.4

References

1. **Mortazavi A, Williams BA, McCue K, Schaeffer L, Wold B.** 2008. Mapping and quantifying mammalian transcriptomes by RNA-Seq. Nat Meth **5:**621-628.
2. **Benjamini Y, Yekutieli D.** 2001. The control of the false discovery rate in multiple testing under dependency. **29:**1165-1188.

SUPPLEMENTAL MATERIALS

Table S1. Primers used for gene disruption and site-directed mutagenesis.

Name	Sequence
LDH-HP1	AAATATTTTTAGTAGCTTAAATGTGATTCAACATCACTGGA GAAAGTCTTGTGTAGGCTGGAGCTGCTTC
LDH-HP2	ATTGGGGATTATCTGAATCAGCTCCCCTGGGTTGCAGGGGAG CGGCAAGAATTCCGGGGATCCGTCGACC
LDH-U	AAATATTTTTAGTAGCTTAA
KANk2- k2	CGGTGCCCTGAATGAACTGC
FRDhf-NEW-HP1	CTTACCCTGAAGTACGGGGCTGTGGGATAAAAACAATCTG GAGGAATGTCGTGTAGGCTGGAGCTGCTTC
FRDhr-NEW-HP2	CCATACAAAACGGCCCGCCATAGGCGGGCCGGATTTACATTG GCGATGCGATTCCGGGGATCCGTCGACC
FRD-U	CTTACCCTGAAGTACGGGGC
rbcL-K198G-F	GGACTTCACCGGTGACGACGAAAACATCAACTCGC
rbcL-K198G-R	AGACCGCCGCGCAGACAT
rbcL-D200G-E201G-F	CACCAAAGACGGTGGTAACATCAACTCGCAGCCGTTCCAAC
rbcL-D200G-E201G-R	AAGTCCAGACCGCCGCGC
rbcL-K172G-F	TTGCACGATCGGTCCAAAACTCGGTCTGTCGGCG
rbcL-K172G-R	CCCAGCATCGGACGGCCG
rbcL-K331G-F	CGTCGTCGGCGGTCTGGAAGGCGAC
rbcL-K331G-R	GTGCCGGAGTGGAGGTGG

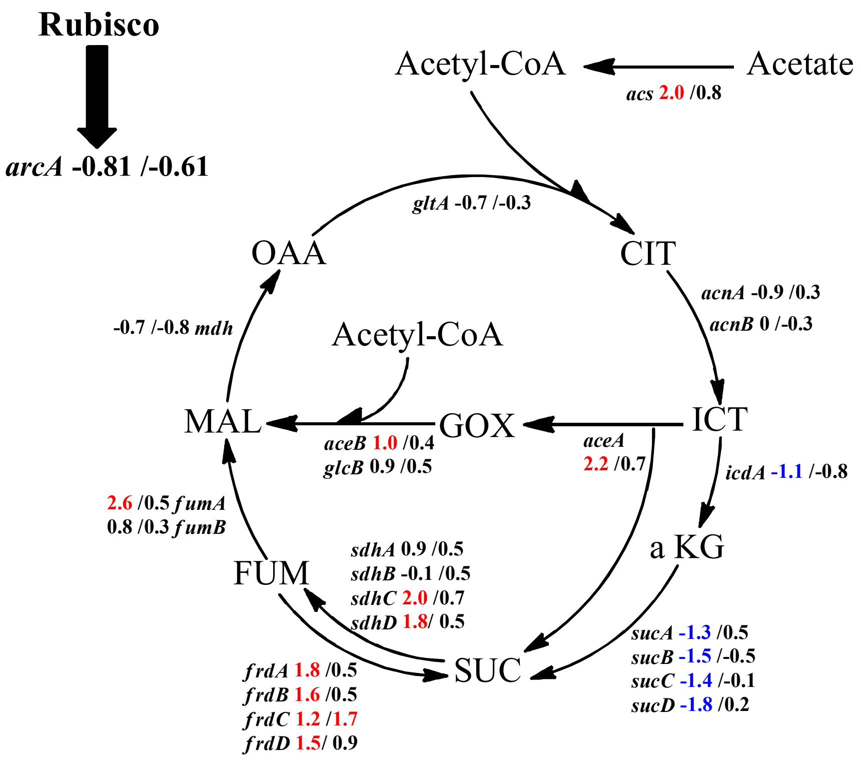

Fig. S1 The transcriptome of *E. coli* strains J3 (first value) and JB (second value) regarding the glyoxylate shunt. Values for each gene are log_2Ratio that are defined in Text S1. All log_2Ratio reported here are statistically significant where all p-values are less than 1×10^{-3} and all False Discovery Rates (FDRs) are less than 0.05. The pathway was constructed based on KEGG (Kyoto Encyclopedia of Genes and Genomes) database. Abbreviation: CIT, citrate; ICT, isocitrate; GOX, glyoxylate; α-KG, α-ketoglutarate; SUC, succinate; FUM, fumarate; MAL, malate; OAA, oxaloacetate.

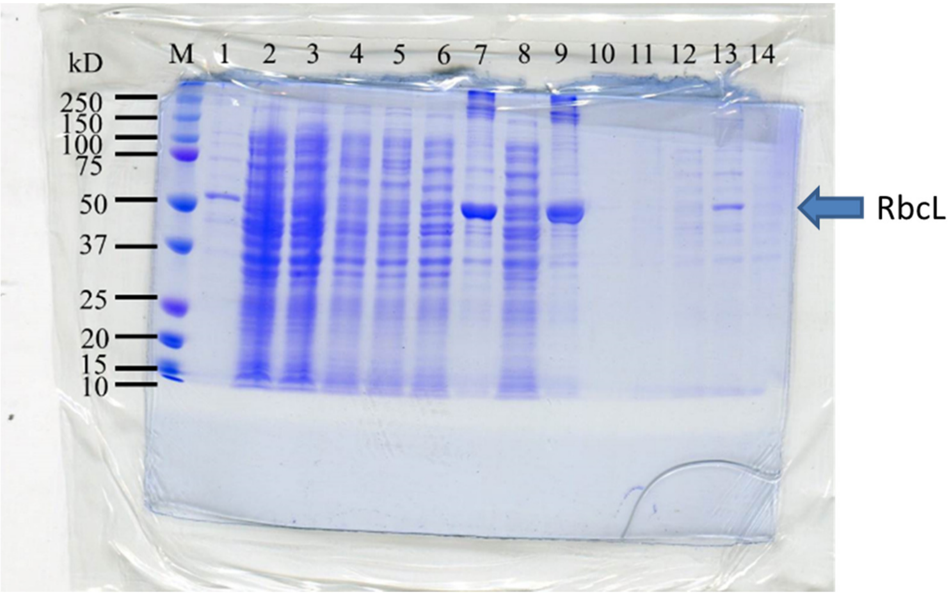

Fig. S2 SDS-PAGE for confirming the overexpression of Rubisco (K198G). M, Marker; lane 1, MZLF3(K198G)+IPTG (supernatant); lane 2, *E. coli* BL21(DE3) (whole cell); lane 3, *E. coli* BL21(DE3)+IPTG (whole cell); lane 4, MZLF (whole cell); lane 5, MZLF+IPTG (whole cell); lane 6, MZLF3 (whole cell); lane 7, MZLF3+IPTG (whole cell); lane 8, MZLF3(K198G) (whole cell); lane 9, MZLF3(K198G)+IPTG (whole cell); lane 12, MZLF3+IPTG (supernatant); lane 13, MZLF3+IPTG (supernatant); lane 14, MZLF3(K198G) (supernatant).
